# Timing anticipation in adults and children with Developmental Dyslexia: evidence of an inefficient mechanism

**DOI:** 10.1038/s41598-020-73435-z

**Published:** 2020-10-15

**Authors:** Elena Pagliarini, Lisa Scocchia, Elisa Granocchio, Daniela Sarti, Natale Stucchi, Maria Teresa Guasti

**Affiliations:** 1 DiSLL Dipartimento di Studi Linguistici e Letterari, Università degli Studi di Padova, Via E. Vendramini, 13, 35137 Padua, Italy; 2 Department of Psychology, Università degli Studi di Milano-Bicocca, Piazza dell’Ateneo Nuovo, 1, 20126 Milan, Italy; 3 Developmental Neurology Unit, Fondazione I.R.C.C.S. Istituto Neurologico Carlo Besta, Via Giovanni Celoria, 11, 20133 Milan, Italy

**Keywords:** Human behaviour, Dyslexia

## Abstract

Developmental Dyslexia (DD) is a learning disorder characterized by specific difficulties in learning to read accurately and fluently, which has been generally explained in terms of phonological deficits. Recent research has shown that individuals with DD experience timing difficulties in the domains of language, music perception and motor control, probably due to impaired rhythmic perception, suggesting that timing deficit might be a key underlying factor to explain such a variety of difficulties. The present work presents two experiments aimed at assessing the anticipatory ability on a given rhythm of 9-year old Italian children and Italian adults with and without DD. Both adults and children with DD displayed a greater timing error and were more variable than controls in high predictable stimuli. No difference between participants with and without DD was found in the control condition, in which the uncertain timing of the beat did not permit the extraction of regularities. These results suggest that both children and adults with DD are unable to exploit temporal regularities to efficiently anticipate the next sensory event whereas control participants easily are. By showing that the anticipatory timing system of individuals with Developmental Dyslexia appears affected, this study adds another piece of evidence to the multifaceted reality of Developmental Dyslexia.

## Introduction

Learning to read involves establishing connections between graphemes (printed words) and phonemes (sounds). Generally, children are taught to read starting from 6 years of age (Italian mainstream) and the full mastery of reading requires some years of practice and instruction. Once completely automatized, reading becomes fluent and does no longer require conscious control. However, the 3–7% of the school population struggles in automatizing the reading process. Indeed, these children suffer from Developmental Dyslexia (henceforth DD). In Italy, the percentage ranges from 1.5 to 5%^1–4^. Children with DD turn out to read slower and less accurately than children of equal age who received the same amount of education. The Diagnostic and Statistical Manual of Mental Disorders (DSM-5) describes Developmental Dyslexia as “a pattern of learning difficulties characterized by problems with accurate or fluent word recognition, poor decoding, and poor spelling abilities”^5^ (p. 67) in children with average intellectual abilities, without mental or neurological disorders, proficient in the language of academic instruction and provided with adequate educational instruction. Developmental Dyslexia is thus included among the specific learning disorders.

Among the numerous theories seeking to explain Developmental Dyslexia, the Phonological Theory, according to which the core problem is due to phonology related processes^6–8^, enjoys wide agreement. Children, who turn out to be affected with DD usually show a phonological deficit before they learn to read and its severity is usually predictive of the variation in the severity of the reading deficit. By consequence, children with DD struggle with reading accurately and fluidly and experience particular difficulties in phonological awareness tasks (explicit phonology), in phonological processing tasks and in tasks demanding rapid automatized naming (implicit phonology). As a quick overview, children with DD have been found to perform more poorly than younger reading age matched children in the selection of the word out of a sequence of four words that did not rhyme^9^; in syllable segmentation^10^; in nonwords repetition^11^, in rapid automatized naming (RAN)^12^.

Beside the undisputed core phonological deficit, previous research has shown that other areas are also compromised, such as core language abilities and language processing^13–21^, auditory^22,23^ and visual^24–26^ perception, and fine and gross motor skills^27–29^ that also extend to difficulties in handwriting^27,30–32^, leading to the proposal of different theories of dyslexia. More recent research focused on the relation between dyslexia and rhythmic abilities. Rhythm is, broadly speaking, a series of sounds and silences perceived as temporally organized. To have a rhythm, a basic periodic pulsation (tactus, meter, or metronome) is a necessary but not a sufficient condition. At the very least, a rhythm requires also another level of temporal organization^33–35^, which allows to decide when to act, that is to *anticipate* a future event. Consequently, at least two basic skills are required to produce a rhythmic behaviour: synchronizing to a given meter (and thus the ability to extract a regularity) and anticipating future events, that is to say to establish when something is going to occur and being prepared to act. Previous research has observed that individuals with DD experience difficulties in rhythmic perception in the music domain^36–41^ and in some rhythmic aspects of the language domain, such as stress detection^37,42^. Some of the studies inspired by the idea of a rhythmic impairment have focused on discrimination of the rise time of a sound, a feature of a sound allowing timbre discrimination (in music) or phonemes discrimination (in speech perception)^43,44^, revealing that children with DD are impaired—across languages having different rhythmic structure—in discriminating amplitude envelope rise time (English^43,45^; Chinese and Spanish^45^; Hungarian^46^; Finnish^47^; French^40^). There is also increasing evidence that rhythmic abilities are associated to phonological awareness, an essential precursor of reading^36,38,48–50^. The link between rhythm and phonological awareness emerges also in studies showing that music and rhythmic training improves phonological awareness and reading skills^48,51–55^. Rhythm facilitates segmentation of the continuous speech into units of different sizes, phrases, words, phonemes and contributes to the recovery of phonological words, as shown in several studies on infants^56–61^. Thus, rhythm can help children in building their phonological lexicon that can be then used to build an orthographic lexicon when children learn to read. Difficulties with rhythm may impact on the development of the phonological lexicon, of grammar^56,62^ and then on reading, as proposed—among others—by the Temporal Sampling Theory^44^. Difficulties with rhythm may impact on handwriting and even in this case, phonology may play a mediating role^63^. Another component of rhythmic behaviour investigated in dyslexics is sensorimotor synchronization^64^, mostly tested by tapping in time to a metronome beat^41,65,66^. Adults with DD differed from their control in precision, that is in the variability of the inter tap interval (ITI) (the ITI is measured as the difference between the expected ITI and the observed ITI), but did not differ in the ITI per se^66^. Instead, significant differences in the ITI have been observed between a group of children with DD and a control group with very fast tempos (120 and 150 bpm), but not in a slower tempo (90 bpm)^41^, as well as in precision. Furthermore, there is evidence showing that individual differences in sensorimotor synchronization are related to individual differences in reading and spelling tasks^36^. Two studies^67,68^ combining behavioral and neural measurements showed that the timing representation itself is deficient in dyslexics, ruling out the possibility that impairments in the domain of sensorimotor synchronization are due to an independent motor deficit. The first relevant study is a work by Colling et al.^67^ which studied children’s neural and behavioural entrainment to auditory stimuli. Differently from previous tasks in which participants are asked to tap to any beat, in this study participants were instructed to tap along with every second beat of the auditory stimulus. Both the dyslexic and control children were trained to tap on every second beat until they were equally proficient. No significant differences in the tapping behaviour were found between the two groups. However, the EEG results revealed differences in terms of preferred phase, due to children with DD having a preferred phase synchronizing ahead of the beat for both the tapping conditions and an auditory control condition. These results suggest that prediction measured through synchronization is reduced in dyslexia because a predictable periodic template is not established, and therefore—as the authors suggested—the problem may lie in perception and not production. The second relevant study is a work by Soltész et al.^68^. Dyslexic adults were asked to detect occasional white noise targets interspersed with tones occurring regularly at two different isochronous rates (2 Hz or 1.5 Hz). Dyslexic adults were equivalent to controls for the behavioural task and yet showed neural differences. The strength of phase locking and the contingent negative variation amplitude 30 ms before the occurrence of the beat were both significantly weaker in dyslexics, suggestive of weaker entrainment and less preparatory brain activity (the contingent negative variation is an event-related component that reflects anticipatory attention and motor preparation in anticipation of the forthcoming stimulus^69^). Moreover, the authors of this study found that the pre-stimulus delta phase angle (− 2 ms) of the target trials was predictive of the reaction time of the control group, but not of the dyslexic group, despite their equivalent behavioural performance. Importantly, both the contingent negative variation amplitude and phase locking strength significantly predicted sensitivity to phonological awareness measures and reading measures, suggesting a functional link between neuronal anticipatory entrainment in the delta frequency range and reading performance. To sum up, previous studies showed that the sensorimotor coupling is generally well-preserved in the dyslexic population (though sometimes dyslexics are less precise than controls in tapping), but that neural rhythmic entrainment is atypical in individuals with dyslexia. How can we explain this discrepancy between the behavioural and the neural outcomes? One hypothesis is that dyslexics have a well-compensated synchronization behaviour. An alternative hypothesis, that we explored in this study, is that dyslexics have problems with the other skill needed to produce a rhythmic behaviour, which has not been studied yet, that is anticipation. In fact, not only does rhythm lead to synchronize our behaviour with a periodical pulsation but, as we said earlier, thanks to its temporal organization it also allows to decide when to act, that is to anticipate a future event and to activate the motor preparation for taking effective action.

Our work aimed at testing the hypothesis that individuals with DD experience specific difficulties with anticipation. To test this hypothesis, we engaged well compensated adults and children with DD along with two control groups (a group of typically developed adults and a group of typically developed children, TD) in a task measuring anticipation skills through a warning and imperative paradigm^69^. During the familiarization phase, participants heard a regular sequence of beats (80 bpm) designed to allow the generation of an abstract temporal representation of the heard sequence. In the testing phase, they were tested on the same sequence of beats with the exception that couples of randomly distributed adjacent tones (warning beat (WB) and imperative beat (IB)) were auditorily different from the others (the first overtone—880 Hz—was added to the basic sound used to present the rhythmic pattern). The role of the first tone of the couple (warning beat, WB) was that of alerting participants, that is, putting them in an anticipatory modality (i.e., to be ready to act), as it cued the arrival of the second (imperative beat, IB). Participants were told that at the WB they did not have to do any action, but had to prepare for acting. At the IB, participants had to give a single response by tapping simultaneously with it. By singling out couples of beats, we were able to introduce some minimal and simple temporal organization in our regular sequence of beats, which is required to obtain a rhythm. In this way, we were able to investigate whether participants could exploit the cue from the WB to decide *when* to act. Therefore, participants were expected to anticipate the upcoming input on the basis of the given rhythmic structure and were expected to give their single response exactly *when* the IB took place. In other words, the WB-IB couple of beats made it possible to investigate anticipation skills, because we used the WB to promote an anticipatory behavior, i.e., to prepare the participant to tap to the IB. So, the novelty of our task lies in the possibility to directly test anticipation, based on the ability of extracting the regularity of the sequence, which is then used to timely tap on the IB just after the WB—differently from a tapping task—where participants are required to give multiple serial responses by tapping in synchrony with a metronome and therefore measures if participants are or not in synchrony.

Adults were tested in three conditions: the Unstressed condition (condition 1), which was a plain metronome pulse; the Stressed condition (condition 2), which consisted of an alternation of strong and weak beats; the Unpredictable condition (condition 3), which served as a control condition since it consisted of an unpredictable sequence of beats. Children were only tested in the Unstressed condition (condition 1) and the Unpredictable condition (condition 3). Details for each conditions are reported in Fig. 4. For each condition, ten WB-IB couples were presented, which we will refer to as *Repetitions.*

Regarding the measurements, the accepted behaviour for simple mean reaction time is a delayed response of about 150 ms for sound stimuli^70,71^; any positive error smaller than 150 ms could be interpreted as a measure of some anticipatory behavior. When asking to tap in synchrony with a simple sequence of auditory tones, there is a systematic tendency for tap responses to automatically anticipate the signal by minus 30–50 ms^72^, usually referred to as negative mean asynchrony (NMA). The task we adopted (warning-imperative) is also clearly distinct from a reaction time task. On the one hand, we expected typically developed individuals to tap at the same time with the IB or slightly before as a consequence of the NMA^64,73^. On the other hand, if individuals with DD suffer from an impairment in anticipatory abilities, they may have trouble to prepare themselves and decide when to tap at the IB. We planned to measure the *timing error* (calculated as the difference between the observed time, namely the time of the key press on the IB and the expected time, namely the IB time) and the *individual consistency* (the standard deviation of timing error computed on each set of ten responses for each participant). When referring to our experimental conditions, we expected to find group differences when measuring the timing error between individuals with and without DD in the Unstressed and Stressed Conditions for adults; only in the Unstressed condition for children (since only this condition was tested), but we did not have any specific prediction beforehand on whether DD participants would over-anticipate or would delay the estimation of the occurrence of the IB. We also predicted DD participants to be individually less consistent than the control participants. Importantly, if the group differences were due to the fact that individuals with DD are generally slower than typically developing individuals, we would expect group differences also in the Unpredictable condition, i.e., individuals with DD should respond on average more slowly than controls. On the contrary, if individuals with DD have only poor anticipatory skills, we do not expect any group difference in the Unpredictable condition, as beats being unpredictable, there is no basis for building a temporal representation to exploit during the testing phase.

One ingredient of our task, needed also in the tapping task, is perceptual synchronization, a skill that develops over years and reaches adult-like performance at the age of 6–7^74^. In addition, our task involves a double inhibition component: first, participants needed to refrain from tapping to every beat; second, they have to inhibit their tapping to WB and tap to the IB. That is, our task also involved the inhibition component of executive function skills, which are known to develop over the year even up to 12 years of age^75^. Therefore, we expected children (with and without DD) to display a higher NMA.

We also tested reading abilities (speed and accuracy) both in adults and children, to test potential correlations between anticipation skills and reading abilities. In addition, we measured basic motor skills of DD adults through the Pegboard battery. The fine motor abilities of children were not tested since previous findings in the literature showed that manual dexterity of children with DD does not differ from that of their peers^41^.

## Results

### Reading proficiency (adult participants)

Generalized Linear Model (GLM) analyses with Group (DD, TD) and Gender (M, F) as between-subject factor on reading time (syllable/second) and error scores revealed a main effect of Group for the error scores, *F*(1, 35) = 39.51, *p* < 0.001, η^2^_p_ = 0.53, and for reading time, *F*(1, 35) = 94.4, *p* < 0.001, η^2^_p_ = 0.73. Adults with DD were slower and made more errors than TD adults.

### Reading proficiency (child participants)

GLM analyses were conducted on error scores and reading time (syllable/second) with Group (DD, TD) and Gender (M, F) as between-subject factors, and Task (Words, Non-Words) as within-subject factor. A significant main effect of Group, *F*(1, 43) = 60.4, *p* < 0.001, η^2^_p_ = 0.58 was found for the error score, as DD children made more errors than TD children. For the reading time, the interaction Task by Group was significant, *F*(1, 43) = 37.6, *p* < 0.001, η^2^_p_ = 0.46, as well as the main effects of Group, *F*(1, 43) = 77.6, *p* < 0.001, η^2^_p_ = 0.64, and of Task, *F*(1, 43) = 153.2, *p* < 0.001, η^2^_p_ = 0.78. As expected, DD children read slower than TD children; the interaction was due to the fact that TD children read words faster than non-words (whereas DD children were slow both when reading words and when reading non-words).

### Performance on the Purdue Pegboard Battery (adult participants)

Two separate GLM analyses were performed on the scores of the first three sub-tasks (Right Hand (RH), Left Hand (LH), Both, treated as within-subject factor) and on the last sub-task (Assembly), with Group (DD, TD) and Gender (F, M) as between-subject factor. The first analysis showed a main effects of Group, *F*(1, 35) = 4.93, *p* < 0.05, η^2^_p_ = 0.12, as DD participants inserted fewer pins than TD participants, and a main effect of sub-task *F*(2, 70) = 4.93, *p* < 0.001, η^2^_p_ = 0.46, as the skill decreased from RH to LH. Neither main effects nor interactions emerged from the second analysis on Assembly score. Mean and standard error of the score of each sub-task are reported in Table 1.

**Table 1:** Mean performance and its standard error (in parentheses) in the Purdue Pegboard Battery.

	Adults	
	DD (N = 16)	TD (N = 23)
I. Pegboard RH	13.8 (0.5)	14.9 (0.4)
II. Pegboard LH	12.7 (0.6)	14.3 (0.5)
III. Pegboard Both	11.1 (0.4)	11.9 (0.3)
IV. Pegboard Assembly	31.4 (1.7)	33.2 (1.3)

### Anticipatory timing task (both adult and child participants)

#### Pre-processing of data

In order to detect outliers in each set of repeated responses for the Unstressed (1) and Stressed (2) Conditions, we filtered the data of each participant using the *Median Absolute Deviation* (MAD)^76^ computed on the ten repetitions. Then we calculated the z point corresponding to each response *x*_*i*_:

$${z}_{i}=\left|\frac{0.6745 \cdot ({x}_{i}-M)}{MAD}\right|$$

where M stands for median. When z was > 2, the data point was replaced by the median. As a result, the 4% of the adult responses and the 4.4% of children responses were substituted. We did that because the warning-imperative task is very demanding and a momentary drop of attention can produce clearly aberrant responses. Thus, this preliminary procedure of filtering data was meant to exclude these very few meaningless responses (for instance, errors greater than 750 ms or smaller than − 250 ms). The data of the Unpredictable condition were left unfiltered given their expected great variability. Afterwards, also the presence of possible outliers among the participants was checked. Participants with a mean error (computed on the Unstressed (1) and Stressed (2) Conditions) outside the interval delimited by ± 3SD of participants’ mean were considered outliers. As a result, one participant of the adult TD group was discarded. Two dependent variables were considered for the analysis: the *timing error* and the *individual consistency*.

### Timing error

In order to determine whether the timing of tapping was synchronous with the occurrence of the IB beat, the timing error was calculated as the difference between the observed time, namely the time of the key press on the IB and the expected time, namely the IB time. Thus, positive errors represent delayed responses; negative errors represent anticipated responses; zero errors represent flawless simultaneity. Timing error was calculated for each condition.

#### Adults

**Figure 1:**
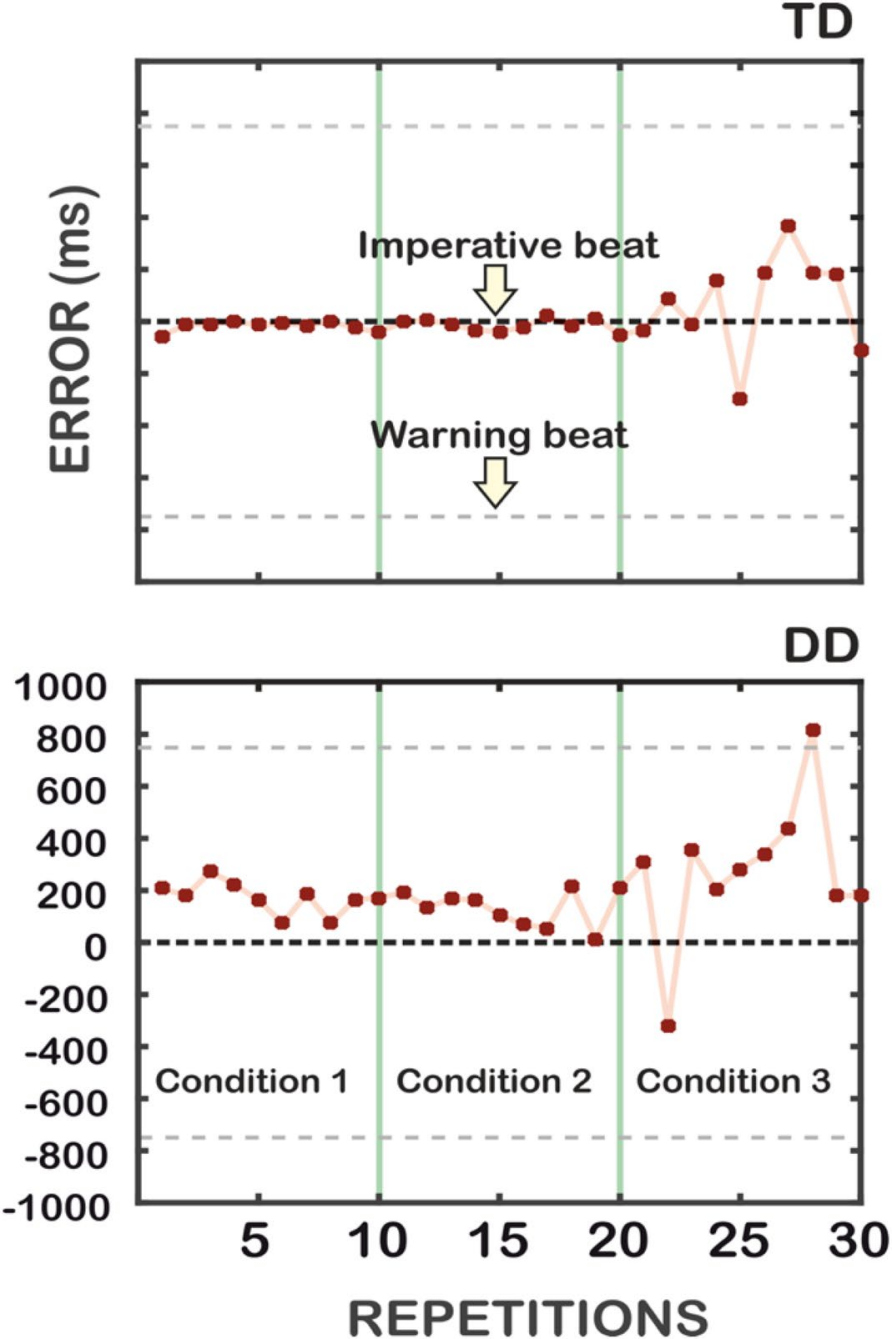
Timing error of a TD (upper panel) and of a DD participant (lower panel), adult participants. On the X-axis, the 30 repetitions of IB (ten for each condition) are reported. On the Y-axis, the timing error (in ms) is reported. Time zero represents the time of occurrence of the IB (corresponding indeed to the expected response). Red markers stand for observed responses. Positive errors represent delayed responses; negative errors represent anticipated responses; zero errors represent flawless synchrony. Green vertical lines separate the three conditions.

Figure 1 displays the performance of typical adult participants. As displayed in Fig. 1, the TD participant showed good predictive skills. In the Unstressed (1) and the Stressed (2) Conditions, adults with TD responded in synchrony with the IB (in sporadic cases the response had few milliseconds of anticipation). On the contrary, adults with DD tended to give a delayed response to the IB and tended to be very variable. In the Unpredictable condition (3), which is meant to be a control condition, participants of both groups were not able to anticipate the IB (as there was no regularity). Both the DD and the TD participants tended to respond after having heard the IB. This finding supports our hypothesis that individuals with DD struggle in temporal predictions and are not merely slower than controls.

**Figure 2:**
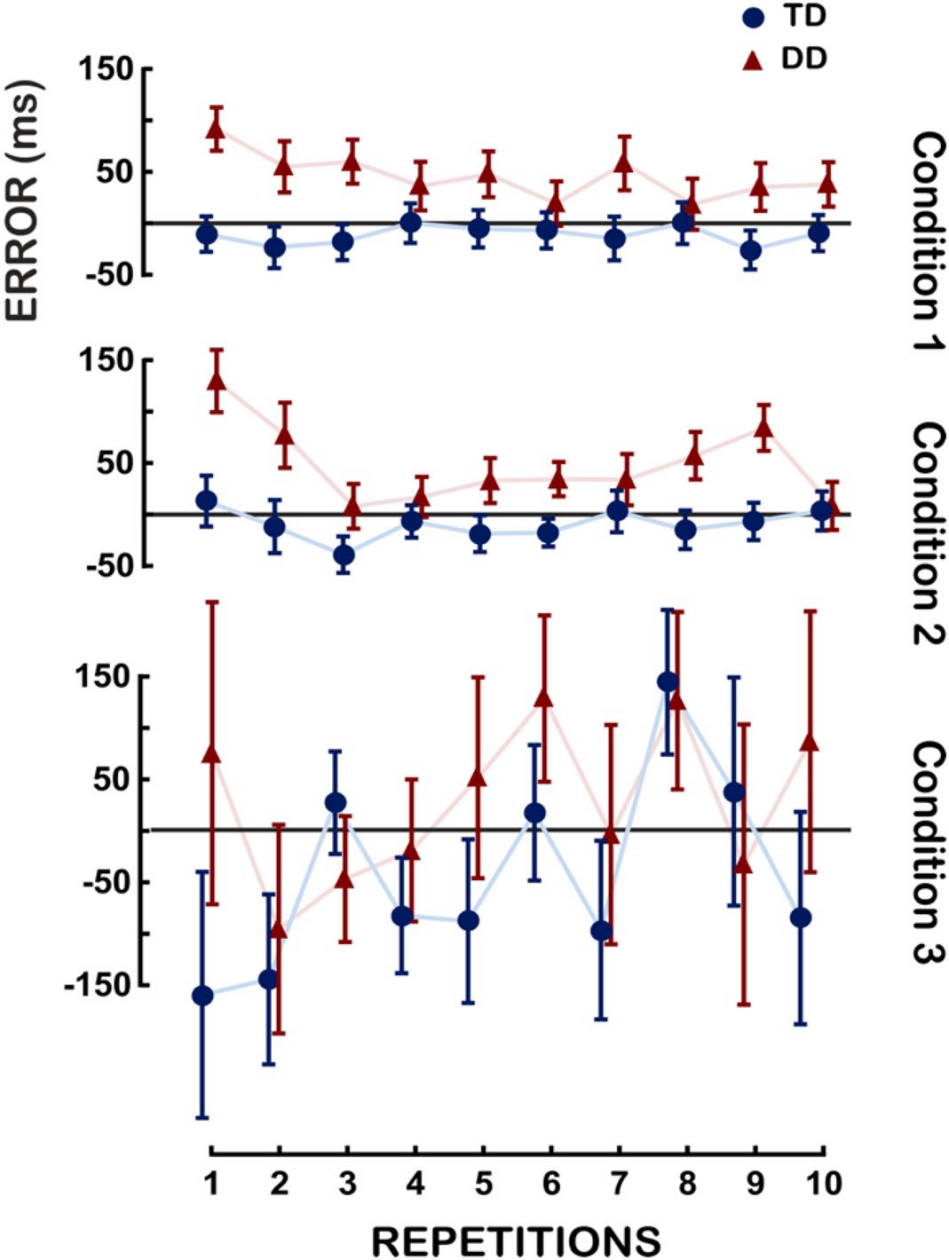
Timing error of the Unstressed (Condition 1), the Stressed (Condition 2) and the Unpredictable (Condition 3)—adult participants. The interaction Group × Repetition is reported for the three experimental conditions. On the X-axis the mean of the ten repetitions is reported. On the Y-axis the timing error (which is calculated as the difference between the observed time, namely the participant’s response, and the expected time, namely the IB) is reported. Vertical bars represent the Standard Error.

GLM analyses on the timing error were carried out for each condition, with Group (DD, TD) and Gender (M, F) as between-subject factors, and Repetition as within-subject factor. Results are shown in Fig. 2 and reported in Table 2.

**Table 2:** Means of timing error (ms) is reported for the Unstressed (condition 1), Stressed (condition 2) and Unpredictable (condition 3) conditions (standard error is reported into brackets).

Group	Adults		Children	
	DD (N = 16)	TD (N = 23)	DD (N = 18)	TD (N = 29)
Unstressed (condition 1)	46 (18)	− 11 (15)	46 (15)	− 40 (12)
Stressed (condition 2)	44 (16)	− 9 (13)	Not administered	Not administered
Unpredictable (condition 3)	24 (44)	− 37 (36)	− 22 (18)	− 59 (14)

In the Unstressed condition (Condition 1), a main effect of Group was found, *F* (1, 36) = 5.89, *p* < 0.05, η^2^_p_ = 0.14. As illustrated in Fig. 2, TD participants were synchronous or slightly anticipated the IB whereas participants with DD were delayed with reference to the IB timing. In the Stressed condition (Condition 2), we also found a main effect of Group, *F*(1, 36) = 6.91, *p* < 0.05, η^2^_p_ = 0.16. As displayed in Fig. 2, participants with DD showed a similar tendency as for the Unstressed condition. Repetitions was also significant, *F*(9, 315) = 4.39, *p* < 0.001, η^2^_p_ = 0.11. Bonferroni post-hoc comparisons showed that the timing error of the first repetition was bigger than the timing errors of the third, fourth and fifth repetition. The interaction, Group x Repetition was significant, *F*(9, 315) = 2.27, *p* < 0.05, η^2^_p_ = 0.06, though Bonferroni post-hocs revealed no significant comparisons. The interaction Gender × Repetition was also significant, *F*(9, 315) = 2.35, *p* < 0.05, η^2^_p_ = 0.06, although again no statistical significant post-hoc comparisons were found. In the Unpredictable (3) condition, no significant difference was found.

#### Children

**Figure 3:**
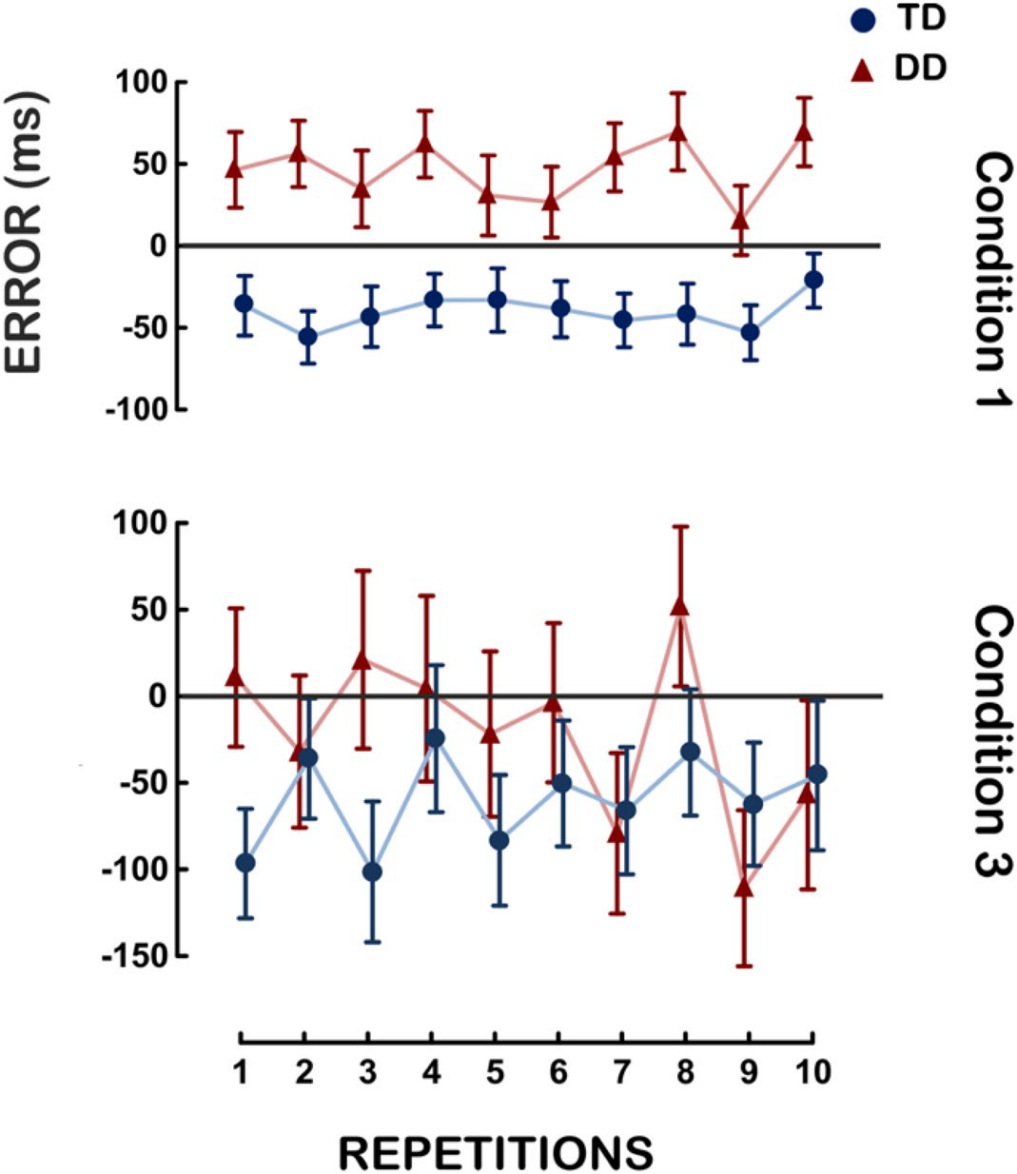
Timing error of the Unstressed (condition 1) Unpredictable (condition 3) conditions—child participants. The interaction Group × Repetition is reported for the two experimental conditions. On the X-axis the mean of the ten repetitions is reported. On the Y-axis the timing error (which is calculated as the difference between the observed time, namely the participant’s response, and the expected time, namely the IB) is reported. Vertical bars represent the Standard Error.

The average performance of the Unstressed (condition 1) and the Unpredictable condition (condition 3) is shown in Fig. 3 and Table 2. The results showed that children’s behavior is similar to the adults’ one. The same GLM analyses on the timing error used for adults were carried out for children. In the Unstressed condition (condition 1), a main effect of Group was found, *F*(1, 43) = 20.8, *p* < 0.001, η^2^_p_ = 0.33. As illustrated in Fig. 3, TD participants anticipated the IB whereas participants with DD were delayed with reference to the IB timing. The interaction Group by Gender by Repetition was significant, *F*(9, 387) = 2.1, *p* < 0.05, η^2^_p_ = 0.05. This two-ways interaction is difficult to interpret given the weak η^2^_p_ and that Bonferroni post-hocs revealed only 4 significant comparisons out of 780. In the Unpredictable condition (condition 3), no significant difference was found except the interaction Group by Gender, *F*(1, 43) = 4.4, *p* < 0.05, η^2^_p_ = 0.09, though Bonferroni post-hocs revealed no significant comparisons.

### Individual consistency

This measure characterizes an individual’s coherence of IB tapping response across the ten repetitions. Individual consistency was measured by calculating the standard deviation of timing error computed on each set of ten responses for each participant.

#### Adults

The GLM analysis on the Unstressed condition (condition 1) with Group (DD, TD) and Gender (M, F) as between subject factors revealed a main effect of Group, *F*(1, 35) = 26.4, *p* < 0.001, η^2^_p_ = 0.43, as participants with DD were less consistent within their tapping response than TD participants (71 vs. 35 ms). Gender was also significant, *F*(1, 35) = 6.1, *p* < 0.05, η^2^_p_ = 0.15, as female participants were less consistent than male participants (61 vs. 44 ms). As for the Stressed condition (condition 2), the GLM analysis with Group (DD, TD) and Gender (M, F) as between subject factor revealed a main effect of Group, *F*(1, 35) = 18.1, *p* < 0.001, η^2^_p_ = 0.34. Similarly, to the Unstressed (1) condition, DD participants were less consistent within their tapping response than TD participants (78 vs. 42 ms). We also found a main effect of Gender, *F*(1, 35) = 13.1, *p* < 0.001, η^2^_p_ = 0.27, due to female participants being less consistent than male participants (75 vs. 45 ms). The interaction Gender × Group was also significant, *F*(1, 35) = 5.50, *p* < 0.05, η^2^_p_ = 0.13. Female participants with DD turned out to be significantly less consistent than TD female participants (103 vs. 47 ms), whereas no difference was found between DD and TD male participants (53 vs. 37 ms). Finally, the GLM analysis on the Unpredictable condition (condition 3), with Group (DD, TD) and Gender (M, F) as between subject factor did not reveal any significant difference.

#### Children

The same GLM analyses on consistency (Unstressed (1) and Unpredictable (3) conditions) run on adult data were carried out on child data. A main effect of Group was found both for the Unstressed (1) and the Unpredictable (3) conditions (*F*(1,43) = 17.7, *p* < 0.001, η^2^_p_ = 0.29 and *F*(1,43) = 20.8, *p* < 0.001, η^2^_p_ = 0.33) indicating that DD children were generally less consistent than TD children. No further significant effect or interaction was found.

### Correlation analyses (both adult and child participants)

A correlation matrix among timing error, individual consistency, reading speed, reading errors and Pegboard scores was computed in order to evaluate the relations among different variables. As timing errors we considered the means obtained by collapsing the ten repetition of each participant. Table 3 reports the correlation matrix for adults. Table 4 reports the correlation matrix for children.

**Table 3:** Adult correlation matrix among C1 (Unstressed Condition) and C2 (Stressed Condition) error (means obtained by collapsing the ten repetition of each participant); C1 and C2 consistency (standard deviations of Unstressed and Stressed Conditions over repetitions) and the other measures assessed in this study.

	C1 error	C2 error	C1 consistency	C2 consistency	Reading time	Reading error	Peg RH	Peg LH	Peg both	Peg ass
C1 error	–									
C2 error	0.81***	–								
C1 consistency	0.06	0.15	–							
C2 consistency	0.40*	0.44**	0.36*	–						
Reading time (syll/s)	− 0.37*	− 0.34*	− 0.47**	− 0.33*	––					
Reading error	0.23	0.22	0.40*	0.23	− 0.75***	–				
Pegboard RH	− 0.36*	− 0.25	− 0.10	− 0.02	0.21	− 0.06	–			
Pegboard LH	− 0.47**	− 0.34*	− 0,14	− 0.19	0.34*	− 0.46**	0.44**	–		
Pegboard Both	− 0.43**	− 0.37*	− 0.03	− 0.17	0.26	− 0.20	0.40*	0.39*	–	
Pegboard Assembly	− 0.44**	− 0.43**	0.00	− 0.24	0.18	0.04	0.55***	0.33*	0.43**	–

Pearson correlation coefficients are displayed *p < 0.05; **p < 0.01; ***p < 0.001.

**Table 4:** Child correlation matrix among C1 (Unstressed Condition) error (means obtained by collapsing the ten repetition of each participant); C1 consistency (standard deviations of Unstressed condition over repetitions) and the other measures assessed in this study.

	C1 err	C1 cons	Word reading time	Word reading error	Non− word reading time	Non-word reading error
C1 error	–					
C1 consistency	0.38**	–				
Word reading time (syll/s)	− 0.41**	− 0.44**	–			
Word reading error	0.38**	0.48***	− 0.64***	–		
Non-word reading time (syll/s)	− 0.40**	− 0.40**	0.94***	− 0.60***	–	
Non-word reading error	0.41**	0.50***	− 0.66***	0.88***	− 0.65***	–

Pearson correlation coefficients are displayed *p < 0.05; **p < 0.01; ***p < 0.001.

For both children and adults, timing error and individual consistency score (adults: C1, C2 error; children: C1 error) negatively correlated with reading speed: participants who were less consistent and who had a higher timing error in anticipating the IB also read fewer syllable per second (this holds both for word and non-word reading in children). Furthermore, errors in reading positively correlated with timing error and individual consistency, especially for children, that is children who made more mistakes while reading had a higher timing error and were more inconsistent. The Pegboard scores negatively correlated with the timing error of both conditions in adults (but not with the individual consistency score).

To obtain more information from the adult correlation matrix, we run partial correlations, controlling for motor skills (Pegboard Right; Pegboard Left; Pegboard Both; Pegboard Assembly). The results, reported in Table 5, showed that the individual consistency score of Unstressed condition (1) negatively correlated with reading speed and positively correlated with reading errors. No correlation is found between timing error and reading speed when controlling for motor skills.

**Table 5:** Adult partial correlation matrix among C1 (Unstressed Condition) and C2 (Stressed Conditions) error (means obtained by collapsing the ten repetition of each participant); C1 and C2 consistency (standard deviations of Unstressed and Stressed Conditions over repetitions) and the other measures assessed in this study, controlled for Pegboard Right, Pegboard Left, Pegboard Both, Pegboard Assembly.

	C1 error	C2 error	C1 consistency	C2 consistency	Reading time	Reading error
C1 error	–					
C2 error	0.75***	–				
C1 consistency	0.02	0.14	–			
C2 consistency	0.33	0.36*	0.38*	–		
Reading time (syll/s)	− 0.22	− 0.22	− 0.46**	− 0.29	–	
Reading error	0.08	0.14	0.40*	0.19	− 0.75***	–

Pearson correlation coefficients are displayed *p < 0.05; **p < 0.01; ***p < 0.001.

## Discussion

The current study explored the hypothesis that individuals with Developmental Dyslexia (DD) suffer from an inefficient anticipatory timing mechanism. A growing body of research has observed that individuals with DD have difficulties in time perception in various domains^32,43,66,77^. At the same time, another strand of literature has uncovered associations between musical skills—especially aspects related to rhythm—and phonological awareness in TD individuals^36,38,48–50^. Individuals with DD have also been reported to benefit from training in sensorimotor synchronization to a pulse delivered by a metronome^51^, and more generally from music training^52^. Previous studies focusing on sensorimotor synchronization showed that the sensorimotor coupling is generally well-preserved in the dyslexic population (though sometimes dyslexics are less precise than controls in tapping), but that neural rhythmic entrainment is atypical in individuals with dyslexia^41,65–68^.

The aim of the present work was to explore the hypothesis that individuals with DD suffer from an inefficient anticipatory mechanism. Importantly, in our study we assumed that rhythm consists of a periodic pulsation and at least one further level of temporal organization that allows to orient in time. Therefore, rhythm allows to make temporal predictions, i.e., it can be exploited to decide when to act, that is to anticipate a future event. In line with this hypothesis, we engaged participants in a task in which they were first familiarized with a periodic pulsation and they were subsequently tested by means of the so-called warning-imperative paradigm^69^ that is, in the testing phase, the familiar sequence of beats was presented again to the participants, with the exception that random couples of adjacent beats differed auditorily from the other beats. These couples of beats superimposed a temporal organization to the regular sequence participants were familiarized with. We called the first different beat warning beat (WB) and the second different beat, imperative beat (IB); the WB informed the participant about when the second different beat (IB) was going to occur. In the experiment, participants were asked to tap timely with the IB. The assumption was that participants should effortlessly know when to act, since the WB gave advance notice of the occurrence of the IB. Therefore, we hypothesized that if participants were able to extract the temporal structure of the presented rhythms, they would have tapped on time to the IB or just earlier. Consistent with our hypothesis, both in the Unstressed (1) and Stressed conditions (2), adult controls tapped timely with the IB or slightly before. In contrast to control participants, adults with DD systematically responded with a delay, that is, they showed a tendency for tapping after the occurrence of the IB both in the Unstressed (1) and Stressed (2) conditions. Furthermore, their responses varied more than those of control adults, that is, they were also less consistent than control participants. However, their timing error was generally lower than 150 ms, which is the average reaction time, thus suggesting that adults with DD tried to anticipate the beat, as control participants, even though this resulted in a poor anticipation accuracy. As for the Unpredictable condition (3)—where no regularity was present to enhance the anticipatory behavior—no difference was found between adult controls and adult participants with DD, in line with our hypothesis. This result undermines an explanation often proposed to explain the tendency of well compensated adults with DD (especially in transparent orthography systems as the Italian one) to remain slow in reading. This behavior is often attributed to a domain-general inclination to respond slowly. If this slowness were the cause of their difficulty, we would have expected them to differ from TD individuals in the Unpredictable (3) condition, i.e., they should have answered more slowly than TD participants. In contrast, no group difference emerged.

Children’s results replicated those found with adults. In the Unstressed condition, control children anticipated the IB and tapped generally 30–50 ms in advance, displaying a clear negative mean asynchrony. Children with DD were systematically delayed. As adults with DD, they tried to anticipate the imperative beat, but they had a poor performance. Similarly to adults, children with DD, were less consistent in tapping to the IB than their controls, indicating once again that temporal prediction is challenging for them. In the Unpredictable condition (3), our control condition, no group difference was observed in children as well.

When comparing control children and control adults, it emerged that control children’s negative mean asynchrony was higher than that of adult controls. In the literature, sensorimotor synchronization skills reach adult-like performance at the age of 6–7^74^. Although our children were 9 years old, it is plausible to think that they did not match completely adults’ performance, since our task was not a purely sensorimotor synchronization task, as it involved a double inhibition component: first, participants had to inhibit their tendency to tap to every beat; second, they had to inhibit their tapping to the warning beat and tap to the imperative. We know that inhibition is a complex construct of executive function that develops over the years and some of its components are still developing at age 12^75^.

In conclusion, DD participants showed a tendency for tapping after the occurrence of the IB both in the Unstressed and in the Stressed conditions (this latter only adults), but their response was lower than the average expected reaction time.

For adults, we also included the Pegboard battery, a task meant to measure basic motor coordination. In this task, participants had to insert as many pins as possible in the holes using their right hand, their left hand, and then both hands; they also had to form as many assemblies following a given procedure. We found a significant group difference: adults with DD were less skilled than controls, as they inserted fewer pins than controls. The fact that the performance of the Pegboard can discriminate between individuals with and without DD is consistent with Thomson et al.^66^. These authors also found a marginally significant interaction between hand and group, which was due to the fact that individuals with DD inserted fewer pins than controls with the dominant hand. Although Thomson et al.’s effect is less strong than the one found in our study, it goes in the same direction. We have to notice that Thomson et al., unlike us, did not require their participants to complete the whole Pegboard battery, as they did not include the Assembly sub-task and this could explain the marginal difference between the results of the two studies. Interestingly, we also found several negative correlations between the subtasks of the Pegboard and the timing errors in the two predictable conditions. Those individuals who had a higher timing errors performed worse at the Pegboard subtasks. Contrary to Thomson et al.’s statement according to which the Pegboard does not include a rhythmic component and thus allows to measure motor-coordination, we believe that it does include a rhythmic component, because in order to perform the task efficiently one needs to generate a “motor” temporal representation of the whole action, which consists of a sequence of sub-actions executed in a coordinated way by both hands, and exploits it to anticipate and prepare the following sub-action.

The correlation analyses showed that—for both children and adults—the timing error and the individual consistency score of the predictable conditions negatively correlated with reading speed: participants who were less consistent and who had a higher timing error in anticipating the IB also read fewer syllable per second (this holds both for word and non-word reading in children). Furthermore, errors in reading positively correlated with timing error and individual consistency, especially for children, that is children who made more mistakes in reading had a higher timing error and were less consistent. We also run partial correlations on the adult dataset, controlling for motor skills. The results revealed that the individual consistency score of the Unstressed condition (1) negatively correlated with reading speed and positively correlated with reading errors, whereas the correlation between timing error and reading is not significant when controlling for motor dexterity. This result suggests that the strength between reading and timing error must be reduced by removing the variance attributable to the motor task; nevertheless there is some clear relation between reading and the precision in the ability to anticipate, which will deserve more attention in future studies.

Our results point to the conclusion that well-compensated adults and children with DD have difficulties with the anticipation of temporal events, despite the high predictability of the stimulus, since in our task we could measure the response accuracy to the highly predictable IB after the warning of the WB. Importantly, the no significant group difference in the control condition ruled out the possibility that individuals with DD are generally delayed in their responses. In this condition, the occurrence of the IB was unpredictable, and no regularity could be exploited in order to predict the incidence of the IB.

Our results call into question Tallal’s rapid auditory processing theory^22,23^, according to which, individuals with DD are not able to integrate sensory information that converges in rapid succession in the central nervous system. The theory was based on findings showing that children with DD could discriminate basic acoustic information (tones of 75 ms) on a par with typically developing children when the inter-stimulus-interval (ISI) was 428 ms, but not when the ISI was 150 ms. However, in our experiment dyslexics struggled to anticipate the forthcoming beat even though the onset-to-onset interval was 750 ms (80 bpm, ISI 550), thus suggesting that it is not frequency to be the source of the difficulties.

A compromised anticipation mechanism might result on poorer precision in synchronization tasks (see “Introduction”) and might be the responsible for difficulties in fluent and rapid reading, in language, in musical and motor activities (like handwriting), all events that unfold in time and are based on temporal anticipation. A speculative proposal—which will deserve further attention in the future—is that an anticipation deficit could cause both oral and written language deficits. The anticipation deficit might only extend to some children with a certain subtype of developmental language disorders, or perhaps it might explain only some of the deficits in either DD or developmental language disorders. As for reading, which is one the most affected area in DD, anticipatory skills are of great advantage, since an efficient and fast reader looks ahead from the incoming input and prepares to read the next word while reading the current word, as suggested by evidence showing that the eyes are fixating ten letter ahead with respect to the pronounced word^78^. Evidence going towards the same direction comes from a study by Ozernov-Palchik et al.^49^, who investigated the relation between metrical and non-metrical rhythms and literacy. Ozernov-Palchik et al. found that both metrical and non-metrical rhythms were associated to phonological awareness as measured by letter-sound processing (a reading-related ability), as many authors have already reported. However, they also found that metrical rhythm, but not non-metrical rhythm (typical of speech), uniquely predicted phonological awareness. This association led the authors to conjecture that the link between musical rhythm and reading-related skills is mediated by the ability to predict on the basis of rich contextual structure^79^. This finding suggests that being able to extract a regularity, as instantiated in a metrical rhythm (typical of music) is a prerequisite to process complex rhythms such as those found in language, but it is not per se sufficient. Possibly, the link between rhythm and phonology may be more complex than previously proposed and may be mediated by the ability to predict or anticipate^79^. In a similar vein, Bonacina and colleagues^51^ established that reading in synchrony with a rhythmic accompaniment for 9 biweekly sessions of 30 min each improves reading speed and accuracy in 12 year old individuals with DD. They interpreted their results in terms of facilitation in word processing due to the metrical rhythm superimposed on the verbal materials to be read^80^. Additional support for the potential role of anticipation in reading is the finding that good readers display a larger electrophysiological response to sound presented in predictable contexts than poor readers^81,82^.

As far as we know, this is the first work which explicitly investigates anticipatory skills in Developmental Dyslexia. As such, more than answering questions, this paper leaves many alternatives open. In our task, we tested the ability to anticipate an upcoming input by producing a response to the imperative beat. In turn, this was possible based on a regularity that could be extracted from a perceived regular string. The present experiment leaves unanswered whether the difficulty is in the perception of the rhythm or in the production of rhythm or both, even though previous results suggest that the timing representation itself is deficient in individuals with DD^67,68^. A deficient timing representation might impact anticipatory abilities—as highlighted by our results—while effecting less synchronization abilities. However, as discussed earlier, the response of individuals with DD was lower than a reaction time. This is compatible with the idea that DD participants attempted to anticipate and they were not merely responding after they heard the IB. However, they were not as good as control individuals. This could suggest that they were able to extract an abstract representation of the regularity—probably assisted by the ease of the task—but were not able to use this representation efficiently.

Individuals with DD experience a wide range of difficulties; nevertheless, they mainly struggle in accurate and/or fluent word recognition and with poor spelling and decoding abilities that are the result of a deficit in the phonological component of language. Future studies should investigate the possible connections between anticipation and phonology, which is the domain of greater difficulties attested in individuals with DD, by relating phonological awareness abilities with anticipatory skills, to better understand how a damage on anticipatory abilities could also impact the phonological domain. All these questions are of great interest and motivate the need for more fine-grained future investigation to better characterize the source of difficulty experienced by individuals with DD.

## Methods

### Participants

#### Adults

Sixteen participants diagnosed with Developmental Dyslexia (DD) (mean age = 22.75; SD = 2.83, 6 female) and 23 control participants (TD) (mean age 24.78, SD = 5.93, 10 female) were tested. The two groups did not differ in age (*p* = 0.22). All participants were born in Italy, were Italian monolingual speakers, used Italian as their first oral and written language and were students at the University of Milano-Bicocca. They were all right-handed and with normal hearing. DD participants were recruited through the University Learning Disabilities Centre. They were diagnosed for DD following the Italian standard criteria: scores at or below the 5th percentile (or 2 SD from the mean with respect to age) in two out of six measures, these measures being reading speed and accuracy in reading words, pseudowords, and text^83^; absence of neurological and sensorial disorders; IQ within 1 SD from the mean; adequate socio-cultural opportunities. Therefore, adult participants with DD included in this study received the diagnosis by an authorized clinical institute who followed these criteria prior entering the university. The diagnosis of DD participants was further confirmed at the end of high school (based on reading text). TD participants had no neurological, psychiatric and auditory deficits and no learning disabilities. On the basis of a preliminary interview with the participants aimed at ascertaining their musical competence, one DD participant was excluded. This participant had been played an instrument for 11 years at a semi-professional level. We excluded her, as musical training may enhance rhythmic abilities^52^ and, if our hypothesis is correct, predictive skills.

#### Children

Eighteen participants diagnosed with Developmental Dyslexia (DD) (mean age = 9.84; SD = 1.0, 9 female) and 29 control participants (TD) (mean age 9.67, SD = 0.73, 13 female) were tested. The two groups did not differ in age (*p* = 0.70). All participants were born in Italy, were Italian monolingual speakers and used Italian as their first oral and written language, were all right-handed and with normal hearing. Participants with DD were recruited from three Italian clinical institutions: the *Developmental Neurology Unit* of the *Neurological Institute Carlo Besta*, the *Policlinico of Milan *and the *Centro di Psicomotricità* of Lodi. Dyslexia was diagnosed in accordance with the Italian standard criteria by the qualified teams of each institution involved in the study, which also determined that the DD children had no psychological, neurological or auditory problems, nor did they have Developmental Coordination Disorders. Italian standard criteria for the diagnosis of dyslexia are as above: scores at or below the 5th percentile (or 2 SD from the mean with respect to age) in two out of six measures, these measures being reading speed and accuracy in reading words, pseudowords, and text^83^; absence of neurological and sensorial disorders; IQ within 1 SD from the mean; adequate socio-cultural opportunities. The TD children were recruited from three different schools in Milan and Verona (north of Italy). The tenets of the Declaration of Helsinki^84^ were observed and the study was approved by the Ethics Committee of the University of Milano-Bicocca (protocol 0012673/13 for adults, protocol 0010172/13 for children) and also by the one of Istituto Neurologico Carlo Besta (275/2012 for children).

Before each testing session, adult participants were explained about the purpose and the procedures of the study by the experimenter and signed an informed consent. As for child participants, their parents were fully informed about the study and signed informed consent.

### Materials

#### Testing adults

##### Reading a text

The *Prova di velocità di lettura di brani per la Scuola Media Superiore*^85^ was used to assess reading proficiency. Participants were asked to read out loud the first of the two texts included in the test battery (the text was “Funghi in città”, from Marcovaldo^86^). Reading speed and error score were considered as dependent variables. Reading speed was scored in syllables per second (571 syllables divided by reading time). Error score corresponded to the number of the mistakes made, counted as follows: 1 point for each word read incorrectly (irrespective of the errors made in the same word); 0.5 point for: shift of accent (e.g., cittadìno → cittàdino); self-correction; lexical substitution (e.g. mutamenti → cambiamenti); same error reiterated in a word presented more time in the text (e.g. di → da, da; di → da) ; uncertainty (e.g., desideri → desi-desideri). The maximum time participants were given to complete the test was 4 min.

##### Fine motor abilities

The *Purdue Pegboard Battery*^87^ assessed the fine movements of the fingers, hands and arms and the fine fingertip dexterity required in assembly tasks. The pegboard had two rows of thirty holes and four cups at the top of the board. The two external cups contain 25 pins in each. For right-handed participants, the cup to the right of the center contains 40 washers and the cup to the left of the center has 40 collars (for left-handed the location of these latter cups is reversed). Our test procedure consisted of 4 sub-tasks: I. Right hand (RH); II. Left hand (LH); III Both hands (Both); IV. Assembly (Assembly). In RH sub-task, participants were asked to pick up one pin at a time from the external right-handed cup with the right hand and to place as many pins as possible in the right-handed row, starting from the top hole (the same procedure was followed for the LF sub-task, but pins were picked from the external left-handed cup with the left hand and placed in the left-handed row). In the Both sub-task, participants were asked to use both hands. Each sub-task lasted 30 s. For the RH and LH tasks, the score was the number of pins inserted in the holes whereas for the Both sub-task the score was the number of pairs of pins inserted in the rows. In the Assembly sub-task, participants were asked to form as many assemblies as possible by conforming to the following procedure: “Pick up a pin from the external right-handed cup and place it in the top hole of the right-handed row”. Simultaneously, pick up a washer with the left hand and drop it over the pin. While the washer is placed over the pin, pick up a collar with the right hand and drop it over the washer. Finally, while the collar is dropped over the washer, pick up another washer with the left hand and drop it over the collar”. Thus, participants were required to move both hands at the same time and alternate them. The Assembly task lasted 1 min. The score was the total number of positioned parts. Each test session was preceded by a practice session. Each sub-task was administered only once, contrary to the standard procedure, requiring three repetitions of each sub-task.

#### Testing children

##### Reading words and non-words task

Part 2 and 3 of the *Batteria per la valutazione della Dislessia e della Disortografia evolutiva-2, DDE-2*^88^ were used to measure single word reading. Children were asked to read aloud four lists of words and three lists of non-words that adhere to the phonotactic constraints of Italian. The dependent variables considered were reading speed and errors’ score. Reading speed was expressed in syllables per second (281 for the words and 127 for non-words divided by reading time). The errors’ score corresponded to the number of words and non-words read incorrectly. Self-correction was not counted as a mistake.

### Anticipatory timing task (both adults and children)

**Figure 4:**
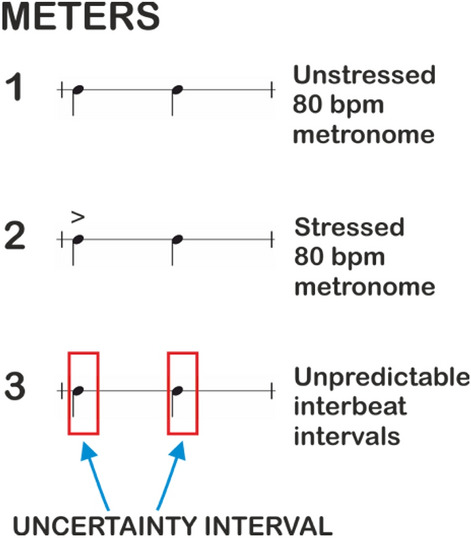
Conditions of the anticipatory timing experiment. Participants were presented two rhythmic sequences and an unpredictable sequence of beats. 1 stands for Unstressed condition; 2 stands for Stressed condition; 3 stands for Unpredictable condition.

We designed an experiment based on the Warning-Imperative Paradigm^69^, in order to assess participants’ ability to generate timing prediction according to a given rhythm and to prepare the motor act. During the familiarization phase, participants listened to a regular sequence of beats. The same sequence was heard in the testing phase, with the exception that randomly there were couples of adjacent beats that were different from the others. In these couples, the first beat was the warning (henceforth WB) and the second was the imperative (henceforth IB) beat. The WB was predictive about the timing of the IB, i.e., when the IB was to come. Three different conditions were always presented in the following order (see Fig. 4):

- *Unstressed condition (1)* It was a plain metronome rhythm, which had a reference tempo of 80 bpm (onset-to-onset intervals of 750 ms). The beats were 440 Hz pure tones with 8 ms rise and fall times and 200 ms steady-state duration. Each couple of WB-IB was randomly presented in a train of 8 beats at random (6 basic tones and a WB-IB couple tones).
- *Stressed condition (2)* It was similar to the Unstressed condition with the only difference that it consisted of an alternation of strong and weak beats. The strong beats were 440 Hz pure tones with 8 ms rise and fall times and 200 ms steady-state duration. The weak beats were 440 Hz pure tones with 4 ms rise and fall times and 100 ms steady-state duration. The intensity of the weak tone was half of the intensity of the strong one. Beats were presented with onset-to-onset intervals of 750 ms. As for the Unstressed condition, WB-IB couples were randomly inserted in sequences of 8 beats (6 basic tones and a WB-IB couple tones).
- *Unpredictable condition (3)* It consisted of an unpredictable sequence of beats. Auditory stimuli were identical to those used in the Unstressed condition. Sounds were presented with a mean onset-to-onset intervals of 750 ms plus an error randomly chosen in an interval of 250 ms. As for the previous conditions, WB-IB couples were randomly inserted in sequences of 8 beats. Given the unpredictable timing, this pattern served as control condition.

Both adults and children were administered this task: adults were tested in all three conditions, whereas children were tested only in the Unstressed condition and the Unpredictable condition, for reason of time and for avoiding loss on attention.

For each condition, the dependent measures were the *timing error* (the difference between the observed time, namely the time of the key press on the imperative beat and the expected time, namely the imperative time), and the *individual consistency* (measured as the variability of repetitions).

**Figure 5:**
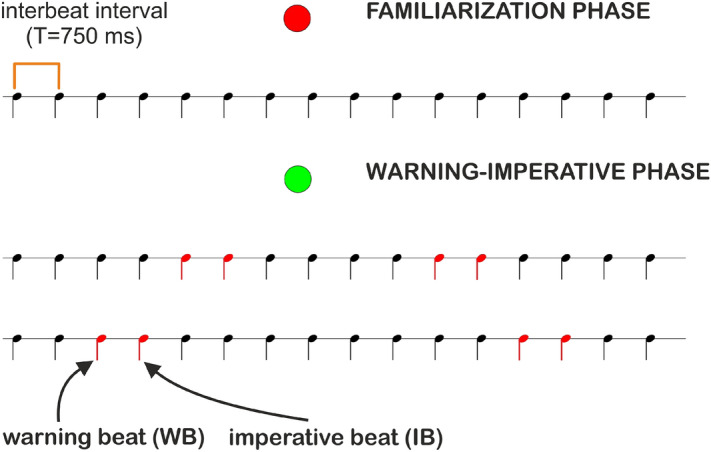
Procedure of the anticipatory timing task. For each condition, participants were presented a familiarization phase (preceded by a red dot), followed by a warning-imperative phase (preceded by a green dot). During the familiarization phase, participants were asked to listen attentively to the sequence (no action was required). During the warning-imperative phase, participants were presented the sequence they were previously familiarized with, and were asked to tap to the left mouse button in sync with the IB. In the figure, the WB and the IB are marked in red for illustration purposes; in the experiment, they were both obtained by adding the first overtone to the basic beat used to present the periodic pattern.

Adult participants were tested individually in a quiet room at the University of Milano-Bicocca, whereas children were tested at the diagnosis centers or at their schools. The total testing session lasted about 30 min, with breaks whenever required. The procedure of the anticipatory timing task is displayed in Fig. 5.

For each condition, participants were first presented a familiarization phase (indicated by a red dot) followed by a warning-imperative phase (indicated by a green dot) (Fig. 5). During the familiarization phase, participants were asked to listen attentively to the given meter (no action was required). During the warning-imperative phase, participants were presented the same rhythmic pattern they were previously familiarized with and were asked to tap to the left mouse button simultaneously with the IB. The interval between the two phases was 3 s. The WB and IB beats were randomly distributed throughout the sequence. In all the conditions, they were obtained by adding the first overtone (880 Hz) to the basic sound used to present the rhythmic pattern. Thus, the WB and IB beats were easily recognizable. In the familiarization phase, one train was presented 2 times per condition; in the warning-imperative phase, one train was presented ten times per condition. In the analyses, the ten WB-IB couples for each condition are referred as *Repetitions*. Before the test session, participants were trained to tap the left mouse button in response to the IB by using a shorter version of the experiment in which only the Unstressed (1) condition was presented. In this version, one train was presented 2 times during the familiarization phase and 5 times during the warning-imperative phase. The experiment was carried out using MATLAB (R2013a) and PsychToolbox_3^89^. All sounds were played via loudspeakers.
